# Plant-RRBS, a bisulfite and next-generation sequencing-based methylome profiling method enriching for coverage of cytosine positions

**DOI:** 10.1186/s12870-017-1070-y

**Published:** 2017-07-06

**Authors:** Martin Schmidt, Michiel Van Bel, Magdalena Woloszynska, Bram Slabbinck, Cindy Martens, Marc De Block, Frederik Coppens, Mieke Van Lijsebettens

**Affiliations:** 1 Department of Plant Biotechnology and Bioinformatics, Ghent University, Technologiepark 927, 9052 Ghent, Belgium; 2 VIB Center for Plant Systems Biology, Technologiepark 927, 9052 Ghent, Belgium; 3 Bayer CropScience N.V., Innovation Center, Technologiepark 38, 9052 Ghent, Belgium

**Keywords:** DNA methylation, Reduced representation bisulfite sequencing, RRBS, *Oryza sativa*, Epiline, Cytosine methylation, Rice, Plant

## Abstract

**Background:**

Cytosine methylation in plant genomes is important for the regulation of gene transcription and transposon activity. Genome-wide methylomes are studied upon mutation of the DNA methyltransferases, adaptation to environmental stresses or during development. However, from basic biology to breeding programs, there is a need to monitor multiple samples to determine transgenerational methylation inheritance or differential cytosine methylation. Methylome data obtained by sodium hydrogen sulfite (bisulfite)-conversion and next-generation sequencing (NGS) provide genome-wide information on cytosine methylation. However, a profiling method that detects cytosine methylation state dispersed over the genome would allow high-throughput analysis of multiple plant samples with distinct epigenetic signatures. We use specific restriction endonucleases to enrich for cytosine coverage in a bisulfite and NGS-based profiling method, which was compared to whole-genome bisulfite sequencing of the same plant material.

**Methods:**

We established an effective methylome profiling method in plants, termed plant-reduced representation bisulfite sequencing (plant-RRBS), using optimized double restriction endonuclease digestion, fragment end repair, adapter ligation, followed by bisulfite conversion, PCR amplification and NGS. We report a performant laboratory protocol and a straightforward bioinformatics data analysis pipeline for plant-RRBS, applicable for any reference-sequenced plant species.

**Results:**

As a proof of concept, methylome profiling was performed using an *Oryza sativa ssp. indica* pure breeding line and a derived epigenetically altered line (epiline). Plant-RRBS detects methylation levels at tens of millions of cytosine positions deduced from bisulfite conversion in multiple samples. To evaluate the method, the coverage of cytosine positions, the intra-line similarity and the differential cytosine methylation levels between the pure breeding line and the epiline were determined. Plant-RRBS reproducibly covers commonly up to one fourth of the cytosine positions in the rice genome when using *MspI-Dpn*II within a group of five biological replicates of a line. The method predominantly detects cytosine methylation in putative promoter regions and not-annotated regions in rice.

**Conclusions:**

Plant-RRBS offers high-throughput and broad, genome-dispersed methylation detection by effective read number generation obtained from reproducibly covered genome fractions using optimized endonuclease combinations, facilitating comparative analyses of multi-sample studies for cytosine methylation and transgenerational stability in experimental material and plant breeding populations.

**Electronic supplementary material:**

The online version of this article (doi:10.1186/s12870-017-1070-y) contains supplementary material, which is available to authorized users.

## Background

In plants, DNA methylation at cytosines occurs in three sequence contexts, i.e. CG, CHG, CHH (H = A, T or C) [1–3], and is regulated by three pathways involving four DNA methyltransferases: the RNA-directed DNA methylation (RdDM) pathway with domains rearranged DNA methylase 2 (DRM2), the chromomethylase 2 (CMT2) and CMT3 pathway and the maintenance methyltransferase 1 (MET1) pathway [4]. The RdDM pathway controls *de novo* DNA methylation via small interfering RNAs (siRNAs) binding specific DNA sequences and guiding DRM2 to initiate methylation of cytosines in all three sequence contexts [5]. CMT3 maintains CHG methylation [6], while CMT2 mediates CHG and CHH methylation through binding to histone H3 lysine 9 (H3K9) methylation [7]. The methyltransferases CMT2, CMT3, and DRM2 redundantly control non-CG methylation, and are components of self-reinforcing loop mechanisms, which include histone H3K9 methylation and siRNAs [7]. MET1 maintains methylation in symmetric CG sites and is independent of siRNAs and histone modifications [8]. In addition to the activity of methyltransferases, the DNA methylation level is also shaped by demethylation processes which can be passive by cell division dilution or active through DNA glycosylases [9].

The methylation levels strongly vary between contexts and species (24 CG, 7 CHG, 2% CHH in Arabidopsis [1] and respectively 86, 74 and 5% in maize [10]). As exemplified in Arabidopsis, intensive methylation in all contexts acts to repress transcription at promoters and transcription start sites (TSSs) of silent genes and at inactive transposable elements (TEs) [11]. Methylated epialleles coincide with gene expression reduction [12–14], whereas cytosine demethylation is accompanied by activation of epiallele transcription [15] or retrotransposition [16]. CG methylation within the gene body of constitutively expressed genes is dispensable in expression regulation [17]. Therefore, DNA methylation has different effects on gene transcription depending on the genomic location and context. In a number of plant species, abiotic and biotic stresses induce changes in the DNA methylation level of specific DNA sequences, resulting in altered expression of stress- or defense-related genes and adaptation to environmental stress (reviewed by [18]). Spontaneous changes in DNA methylation (epimutations) contribute to heritable phenotypic variation [19, 20]. Flowering time and plant height phenotypes that are correlated with distinct cytosine methylation are stably inherited in *Arabidopsis* lines derived from a cross between the wild type and the nucleosome remodeler mutant *ddm1* [21]. *Brassica napus* (canola) epilines have distinct epigenetic signatures of global cytosine methylation, histone H3 methylation and H4 acetylation, and show an enhanced drought stress tolerance, which remained stable for at least seven generations [22, 23].

Although the effects of epigenetic regulation are small compared with those of genetic variation [24], epigenetic breeding is an appealing approach to improve complex traits such as crop stress tolerance and yield stability by selecting putative changes in gene expression at multiple epialleles [25]. Epigenetic breeding requires high-throughput methods for the detection of cytosine methylation to facilitate the identification of individuals with interesting epialleles. In plants, cytosine methylation levels at nucleotide resolution [26] can be evaluated by sodium hydrogen sulfite (bisulfite) conversion-based techniques that are applied in whole-genome bisulfite sequencing (WGBS) using randomly sheared genomic DNA and next-generation DNA sequencing (NGS) [1, 2]. However, profiling methods would be more applicable for large breeding programs where high numbers of individuals are to be tested. Reduced representation bisulfite sequencing (RRBS) has been developed in which methylation-insensitive endonuclease restriction combined with size selection generates specific genome fractions for subsequent bisulfite conversion and NGS [27, 28]. Low cytosine coverage RRBS setups were established in plants, to study methylation in *B. rapa* subgenomes playing an important role in polyploid genome evolution [29] and at *Quercus* gene promoters in response to temperature regimes [30]. Methylation detection was combined with GBS (genotyping by sequencing) resulting in epiGBS, applicable also to non-model plant species lacking a reference genome, allowing to detect methylation polymorphisms from bisulfite-converted samples, but with the need to reconstruct the consensus sequence of the targeted genomic loci [31].

In order to design a high-throughput, cost-effective and reproducible methylome profiling method, we established an efficient workflow for RRBS in plants, referred to as plant-RRBS, using optimized double restriction endonuclease combinations and subsequent bisulfite conversion, followed by NGS and read data processing with conventional bioinformatics programs. The methylation level of tens of millions of cytosine positions was reproducibly detected in multiple biological replicates, which resulted in a broad coverage overlap and allowed the detection of differential cytosine methylation at a thousand CG sites, and less at CHG or CHH sites between lines, i.e. a pure breeding rice line (control) and a derived epiline.

## Results and Discussion

### Plant-RRBS methylome profiling–steps and workflow

**Fig. 1:**
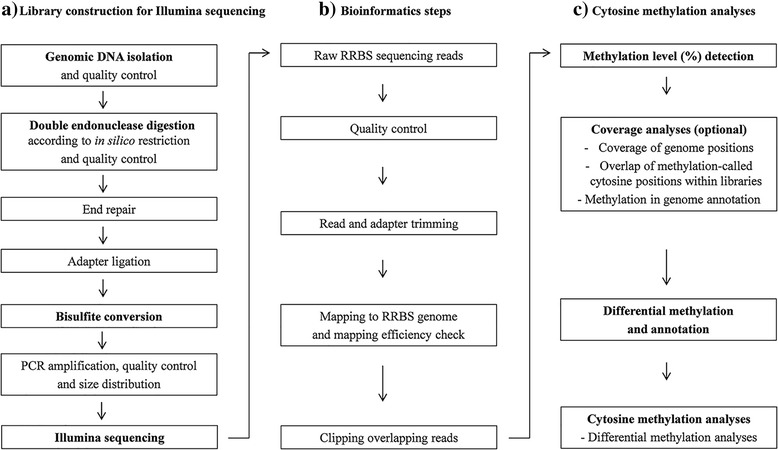
Workflow of plant-RRBS for plant science. (**a**) Main laboratory steps of library construction for Illumina sequencing, (**b**) main bioinformatics steps of plant-RRBS data analysis and (**c**) main steps for determination of coverage and cytosine methylation aspects

A workflow was established for methylome profiling using a rice pure breeding seed lot of an inbred line (named control line) and an epiline, named LR2 with low cellular respiration and high energy use efficiency (EUE; Additional file 1: Table S1). The LR2 epiline was derived from the control seed lot by three selfings combined with testing for cellular respiration (Additional file 1: Table S2) and EUE; the identification and stabilization upon selfing was comparable with the procedure followed in *B. napus* [22, 23, 25]. The major steps in our plant-RRBS methylome profiling include double restriction endonuclease digestion, library construction for Illumina sequencing (Fig. 1a), large data set processing (Fig. 1b) and cytosine methylation detection (Fig. 1c), and are explained below.

#### Effective endonuclease combinations and quality controls

First, genomic DNA was isolated from five individual plants per line and controlled for quality and quantity (Fig. 1a). Restriction endonuclease combinations were selected with cytosine-containing cutting sites that potentially enrich for fragments from C/G-rich regions containing all cytosine contexts (CG, CHG and CHH), aiming to obtain broad coverage of cytosine methylation detection in the plant genome. Appropriate restriction endonuclease combinations have been deduced from *in silico-*simulated complete digestion of the *Oryza sativa ssp. indica* nuclear reference genome that yielded high genome coverage by fragment sizes between 150 and 420 bp, representing the predicted library insert size range. Using *Msp*I (C-CGG) in combination with *Dpn*II (-GATC) or *Ape*KI (G-CWGC), representing an innovative double restriction endonuclease approach in plants, a high *in silico* genome coverage of approximately 37% (*Msp*I-*Dpn*II) or 25% (*Msp*I-*Ape*KI), respectively, was observed in *O. sativa* ssp. *indica*, containing no distinct peaks of satellite DNA or other repeat classes, ideal for effective genome coverage by NGS. Double restriction endonuclease digestions were followed by digestion quality evaluations by gel electrophoresis. A high *in silico* genome coverage was also detected with *Msp*I-*Dpn*II and *Msp*I-*Ape*KI in other plant species with nuclear reference genomes, representing different genome sizes and structural compositions. Indeed, *in silico* digestion of *A. thaliana* (TAIR 10), *Beta vulgaris ssp. vulgaris*, *B. rapa*, *O. sativa* ssp. *japonica*, and *Zea mays* (B73) resulted in respectively 36, 19, 33, 38, and 40% *in silico* genome coverage using *Msp*I-*Dpn*II and 15, 10, 15, 26, and 27% using *Msp*I-*Ape*KI. Hence, the newly proposed combinations *Msp*I-*Dpn*II or *Msp*I-*Ape*KI will be widely applicable for many plant genomes.

#### Library construction for Illumina sequencing and quality control

Upon double digestion, the fragments were end repaired, adapter ligated, bisulfite converted and PCR amplified, resulting in paired-end libraries. Purifications of the libraries were performed using solid-phase reversible immobilization (SPRI), followed by quality control using sub-cloning and sequencing, quantitative PCR (qPCR), and detection of library size distribution with the 2100 Bioanalyzer. The detected library size ranged from approximately 270 to 540 bp (Additional file 1: Fig. S1), containing the insert size range of 150 to 420 bp added with the forward and reverse primer length (sum here 119 bp). The quality-controlled RRBS libraries were sequenced using Illumina HiSeq 2500.

#### Bioinformatics pipeline–Raw read quality check, mapping and efficiency check

The bioinformatics pipeline with all parameters is described in more detail in the Methods section. In a first step, the raw read quality of the different libraries (Fig. 1b) was evaluated with FastQC to ensure read and nucleotide qualities. To ensure identical read lengths, read libraries were trimmed at the 3′ end using the FastX-Toolkit, to limit this confounding variable before starting the downstream bioinformatics pipeline, making direct comparisons between the mapping results possible. With sequencing adapters harnessing the downstream analysis, it was essential to perform adapter trimming, which was done with Trim Galore that by default trims nucleotides with a quality score lower than 20 and discards reads with a length smaller than 20. A low frequency of PCR duplicate reads between 0.08 and 0.76% was detected in the Illumina reads in both the RRBS and WGBS data sets (Additional file 1: Table S3.), thus no need to clean the PCR duplicates from the data sets. The resulting set of high-quality reads was subsequently mapped to the *O. sativa* ssp. *indica* reference genome using BSseeker [32] and bowtie2 [33]. Specific plant-RRBS genome indices were generated, as required by the software, to ensure mapping of the reads obtained from restriction endonuclease fragments. An index was generated for each of the defined cutting sites, C-CGG and -GATC, in the case of *Msp*I-*Dpn*II, and, C-CGG and G-CWGC, in the case of *Msp*I-*Ape*KI. The mapping and its quality was evaluated using Qualimap [34]. Based on the mapping, the calculation of per-base genome coverage, i.e. how many nucleotides in the reference sequence are covered at least once by the set of reads, was performed using BEDTools genomecov [35].

#### Cytosine methylation detection

Cytosine methylation detection is defined as the determination of the methylation level at a cytosine position. The methylation detection was performed for cytosine positions in the reference sequence at which at least ten informative nucleotides (means C or T) were obtained, originating from mapped plant-RRBS reads, representing unconverted and converted cytosines in amplicons of fragments from genomic DNA. Processing of the mapping files was required before cytosine methylation detection, i.e. the obtained BAM files (Binary Alignment/Map, compressed binary version of the Sequence Alignment/Map format) were sorted by coordinate using Picard, and overlapping read pairs were clipped using bamUtil [36] to prevent biased detection. Methylation detection was performed using the well-established BSseeker software [32]. The genomic features were defined as genes, 2.0-kb upstream regions of TSSs (i.e. promoters) or not-annotated regions. The determination of cytosine coverage and methylation aspects were achieved by analyzing cytosine positions having detected methylation levels [26], followed by the determination of the genomic features that overlap with cytosine positions for which a methylation level was detected, and differential cytosine methylation level detection (Fig. 1c). Prior to the detection of differentially methylated cytosine positions, the BSseeker map.gz output files were converted to BED (Browser Extensible Data) format, as required by the often-used methylKit software, using custom scripting. Methylation detection supported by at least ten informative nucleotides (means C or T) at a cytosine position were retained to ensure a certain accuracy of methylation levels in the further analysis. Subsequently, normalization of the libraries was performed using standard parameters of the R package methylKit [37]. The detection of differentially methylated cytosine was performed through methylKit. To speed up NGS analysis, methylation detection, calculation of differential methylation, and determination of genomic features, a high-memory and multi-processor Linux grid server system was used.

### Genome and cytosine coverage aspects of plant-RRBS methylome profiling and WGBS in rice

The *Msp*I-*Dpn*II and *Msp*I-*Ape*KI double restriction endonuclease plant-RRBS setup consisted of biological replicates represented by five individual plants of the rice inbred control or the LR2 epiline. The covered genome fraction, the proportion of covered cytosine positions and the extent of library overlap were analyzed and a comparison between plant-RRBS and WGBS was made.

#### Genome coverage and cytosine coverage in individual plant-RRBS libraries

The average total read number per library was about 58 million paired reads (minimum 15 million paired reads) and read preprocessing and mapping quality of individual libraries are presented in Additional file 1: Table S4. The mapping quality of the different aligned libraries, which is denoted in the quality phred scale and gives the probability of having an incorrect read alignment, was on average 45.3 phred (Additional file 1: Table S4). The genome coverage by at least one read (also denoted per-base genome coverage) was on average approximately 31.0% (Table 1) based on a reference genome size of 427 Mbp [38]. The intersection of detected methylated sites between the individuals per line and per double digest ranges from ~40% for sites covered by all five samples, to ~80% for sites covered by at least three samples (Additional file 1: Fig. S2). This indicates that, in order to achieve a decent coverage of the methylated sites in the genome, the required number of samples should be three or more. The intersection of *in silico* fragments and mapped reads for each individual per line and per double digest varied between 54.84 and 77.72% (Additional file 1: Table S5) which underlines the robustness of our plant-RRBS approach (Fig. 1). Differences between observed and expected suggest reduced efficiency of some of the experimental steps in the procedure, such as double digestion, size selection, adaptor ligation, bisulfite conversion, etc. Their impact on RRBS was investigated in more detail in pigs [39]. A tendency for a higher genome coverage of *Msp*I-*Dpn*II compared with *Msp*I-*Ape*KI, in agreement with the predicted coverage by the *in silico* digestion, is also visualized by the mapped reads of representative samples in the Integrative Genomics Viewer (IGV) at representative genome regions in the coverage data visualization (Additional file 1: Fig. S3). The proportion of covered cytosine positions was up to 48.7% of the genome (Table 1), using a threshold for sufficiently mapped cytosine positions in the reference genome by at least ten informative nucleotides (means C or T). Plant-RRBS generates an effective read number as information resource for broad methylation detection from the analyzed genome fraction (Table 1, Additional file 1: Table S4). Indeed, a maximum of covered cytosine positions of up to 17 million CG sites, 15 million CHG sites, and 56 million CHH sites was detected by the largest library (Table 1, plant 1 of LR2). The coverage varied for both restriction endonuclease combinations with a tendency for higher coverage by *Msp*I-*Dpn*II. *Msp*I-*Dpn*II covered 22.9 to 48.7% of the cytosine positions in the genome which was for the majority of libraries higher than the 24.5 to 34.1% covered by *Msp*I-*Ape*KI (Table 1).

**Table 1:** Genome and cytosine coverage in biological replicates of the control line and the LR2 epiline (fourth selfing) using plant-RRBS, and comparison to WGBS

Line with biological replicates^a^	Restriction endonuclease combination	Genome coverage (%)^b^	Cytosine coverage (%)^c^	Efficiency^d^	Number of analyzed cytosine sites		
					CG	CHG	CHH
					(millions)^e^		
Plant-RRBS							
Control-1	*Msp*I-*Dpn*II	39.6	42.8	1.1	15.3	13.1	48.1
Control-2	*Msp*I-*Dpn*II	42.3	44.9	1.1	15.6	13.6	51.1
Control-3	*Msp*I-*Dpn*II	30.8	32.8	1.1	11.6	10.0	37.0
Control-4	*Msp*I-*Dpn*II	35.0	37.1	1.1	13.0	11.3	42.0
Control-5	*Msp*I-*Dpn*II	21.3	22.9	1.1	8.3	7.1	25.5
LR2–1	*Msp*I-*Dpn*II	46.0	48.7	1.1	16.7	14.7	55.6
LR2–2	*Msp*I-*Dpn*II	45.7	48.5	1.1	16.7	14.7	55.3
LR2–3	*Msp*I-*Dpn*II	27.6	28.8	1.0	9.4	8.6	33.5
LR2–4	*Msp*I-*Dpn*II	45.1	48.4	1.1	17.0	14.7	54.7
LR2–5	*Msp*I-*Dpn*II	41.2	43.9	1.1	15.1	13.2	50.0
Control-6	*Msp*I-*Ape*KI	29.9	34.1	1.1	13.1	11.1	36.6
Control-7	*Msp*I-*Ape*KI	26.2	30.0	1.1	11.7	10.0	31.9
Control-8	*Msp*I-*Ape*KI	25.3	28.7	1.1	11.0	9.5	30.7
Control-9	*Msp*I-*Ape*KI	21.2	24.5	1.2	9.6	8.3	25.9
Control-10	*Msp*I-*Ape*KI	27.8	32.4	1.2	13.0	10.8	34.1
LR2–6	*Msp*I-*Ape*KI	23.0	26.0	1.1	9.8	8.8	27.9
LR2–7	*Msp*I-*Ape*KI	21.8	24.9	1.1	9.5	8.5	26.5
LR2–8	*Msp*I-*Ape*KI	22.3	25.4	1.1	9.6	8.7	27.1
LR2–9	*Msp*I-*Ape*KI	23.2	26.6	1.1	10.2	9.0	28.3
LR2–10	*Msp*I-*Ape*KI	24.0	27.1	1.1	10.0	9.0	29.3
WGBS							
Control −11	-	84.3	52.4	0.6	14.4	15.4	63.9
LR2–11	-	83.7	52.6	0.6	14.5	15.6	63.9

Leaf material from five individual plants per line and per restriction endonuclease combination was used

The bisulfite conversion efficiency rate per biological replicate was higher than approximately 99%

^a^ Name scheme: line–individual plant number (1–11) from selfing generation 4

^b^ Genome coverage: coverage as number of genome nucleotide positions covered by at least one read *100% / 427,026,737 nucleotides in the reference genome [38]

^c^ Cytosine coverage: proportion of analyzed (sufficiently covered) cytosine positions in the genome = sum of analyzed cytosines in CG, CHG and CHH context covered by at least ten informative nucleotides (means C or T) * 100% /178,637,468 cytosines in the reference genome for both strands [38]

^d^ Ratio of cytosine coverage per genome coverage

^e^ Millions of positions of a certain cytosine context (CG, CHG and CHH) in the reference genome for both strands that are sufficiently covered by at least ten informative nucleotides (means C or T) and therefore methylation level of cytosine sites was analyzed [38]

#### Plant-RRBS generates a broad overlap of detected cytosine positions within biological replicates

To investigate whether plant-RRBS generates sufficient overlap in covered regions for differential methylation analysis, we analyzed the cytosine methylation in the overlapping regions between the five biological replicates, i.e. libraries, per line for the two restriction endonuclease combinations (Table 2). The starting point was to determine the number of detected cytosine positions covered by at least one of the five biological replicates of a line, resulting in a total set of positions (union). Thus, the union of covered genome positions was high and up to 54.6% (Table 2, LR2, and *Msp*I-*Dpn*II). The *Msp*I-*Dpn*II combination covered more union positions in a group of biological replicates compared with *Msp*I-*Ape*KI for both control line and LR2 epiline (Table 2). We conclude that plant-RRBS covers in total up to half of the cytosine positions in the rice genome using *Msp*I-*Dpn*II for a group of five biological replicates of a particular line.

**Table 2:** Detected cytosine positions relative to the genome-wide cytosine positions per five biological replicates of the control line and the LR2 epiline (fourth selfing) discriminated by restriction endonuclease combination and cytosine context (CG, CHG and CHH)

Group of biological replicates	Restriction endonuclease combination	Detected cytosine positions in genome			
		CG	CHG	CHH	C (%)
Union (collection of all covered positions in at least one replicate)					
Control	*Msp*I-*Dpn*II	18,507,834	15,887,099	58,570,249	52.0
LR2	*Msp*I-*Dpn*II	19,434,811	16,678,896	61,436,046	54.6
Control	*Msp*I-*Ape*KI	15,715,023	12,901,771	42,018,210	39.5
LR2	*Msp*I-*Ape*KI	14,573,921	12,185,636	39,744,568	37.2
Intersection (common positions in all replicates)					
Control	*Msp*I-*Dpn*II	6,459,314	5,448,976	19,305,085	17.5
LR2	*Msp*I-*Dpn*II	8,023,858	7,435,066	29,372,103	25.1
Control	*Msp*I-*Ape*KI	7,053,953	6,410,114	19,063,516	18.2
LR2	*Msp*I-*Ape*KI	5,454,292	5,509,874	15,910,607	15.0
Jaccard index^a^ (proportion of common on all detected positions)					
		(%)	(%)	(%)	(%)
Control	*Msp*I-*Dpn*II	34.9	34.3	33.0	33.6
LR2	*Msp*I-*Dpn*II	41.3	44.6	47.8	46.0
Control	*Msp*I-*Ape*KI	44.9	49.7	45.4	46.1
LR2	*Msp*I-*Ape*KI	37.4	45.2	40.0	40.4

^a^ Jaccard index or Jaccard similarity coefficient = intersection / union

We proceeded with the detection of common cytosine positions in all five biological replicates of one line, resulting in common sites (intersection), which was relatively high with up to 25.1% commonly covered cytosine positions (up to 44.8 million) in the genome (Table 2). Filtering for common positions in an NGS analysis implies the loss of a fraction of reads but ensures comprehensive and accurate comparison between multiple samples. In conclusion, different aspects of the coverage were determined, showing the fractional enrichment of genome regions by the plant-RRBS method. Commonly occurring cytosine positions can be considered as a measure for the reproducibility of the plant-RRBS method in terms of the detectable proportion of cytosine sites. We conclude that plant-RRBS reproducibly covers commonly up to one fourth of the cytosine positions in the rice genome when using *Msp*I-*Dpn*II within a group of five biological replicates of a line.

Finally, we determined the overlap between libraries in terms of the Jaccard index, which is calculated by dividing the number of commonly covered positions within the libraries by all covered positions, in at least one of the libraries. A major fraction of one third to up to half (33.6***–***46.1%) of the total covered positions was found in the overlap of positions between five libraries per restriction endonuclease combination and per line (Table 2).

#### Comparison between plant-RRBS and WGBS in terms of genome coverage and coverage of cytosine positions

The analysis focused to compare the performance of both methods in detecting cytosine positions with deduced methylation levels, as this is the starting point of the analytic power of bisulfite sequencing-related techniques. Genome coverage is determined by the standard threshold of minimum one mapped read and was applied to the data of both evaluated methods. Plant-RRBS, using reproducible genomic DNA fragments generated by restriction endonucleases, allowed an average genome coverage of 31%, as compared with WGBS using randomly sheared genomic DNA, which covered 84.3 and 83.7% for the control line and the LR2 epiline, respectively (Table 1). The visualization of coverage data of plant-RRBS and WGBS in IGV confirms that those genome coverage percentages extent in representative regions (Additional file 1: Figure S2). The WGBS genome coverage in the control line and epiline is markedly better than the 76% observed in a previous study of an *O. sativa* ssp. indica plant [40]. The per-read cytosine coverage for both RRBS and WGBS indicates that the number of detected cytosine positions and the associated trend line is quite stable when taking positions into consideration that are covered by ten or more reads (Additional file 1: Fig. S4). No large dissimilarity is seen for either RRBS or WGBS, suggesting that the quality for both data sets is comparable. The ratio of cytosine coverage per genome coverage was for WGBS only 0.6 but for RRBS almost two times more efficient (≥1.0). This means that plant-RRBS obtains a better cytosine coverage for a lower genome coverage. Plant-RRBS increases the coverage of cytosine positions detected for their methylation levels with the advantage to analyze a reproducible genome fraction generated by double restriction endonuclease digestion. We conclude that plant-RRBS is beneficial to detect restriction endonuclease-specific genome fractions that are sufficiently covered by the NGS approach (Additional file 1: Figure S4). In consequence, the plant-RRBS produces reads more efficiently and requires much fewer reads for data analysis as compared with WGBS.

### Genomic features at cytosine positions covered by plant-RRBS

We determined the genomic features of covered cytosine positions using the *O. sativa* ssp. *indica* annotation ASM465v1.27 as obtained from Ensembl Plants [41], which does not contain TE annotation features. We performed the annotation of covered cytosine positions on chromosomes of the rice reference genome, determined for gene-associated annotation features. The analysis was performed for both restriction endonuclease combinations and in both lines in all commonly detected CG, CHG and CHH sites of their biological replicates (Additional file 1: Fig. S5). Approximately 45 to 50% of detected cytosine positions were localized in not-annotated regions. A high percentage of approx. 35% of detected cytosine positions was localized in promoters, defined as 2000 nucleotides upstream of the TSS, as compared with protein-coding genes (approx. 15 to 20%). LR2 *Msp*I-*Ape*KI common cytosine sites contained the highest percentage of protein-coding gene positions, including all detected annotation feature subclasses. Differences in percentages of annotated positions were detected for the two restriction endonuclease combinations, and the number of covered cytosine sites was different because the endonuclease restriction combinations cut specific genomic regions. In addition to the genome-wide determination, the visualization of coverage data in IGV indicates those aspects in representative regions (Additional file 1: Figure S3).

Despite current approaches, repeats collapse in reference genome sequences generated by NGS, due to limitations e.g. in read length and assembly procedure of sequence-similar and high-copy DNA elements, allowing very limited determination of repeat annotations [42]. Nonetheless, when additional sequence and annotation information of the genomic features become available, these data can be processed in the presented plant-RRBS data analysis pipeline for this research field. Currently, detailed cytosine methylation of repeats can be analyzed by cloned bisulfite-converted PCR products of repeat elements [43].

In summary, cytosine methylation levels in the individual biological replicates were detected for 22.9 up to 48.7% of the genome-wide cytosine positions, and tens of millions of cytosine positions in common between the five biological replicates of a particular line were subsequently annotated.

#### Cytosine methylation levels at genomic features

**Fig. 2:**
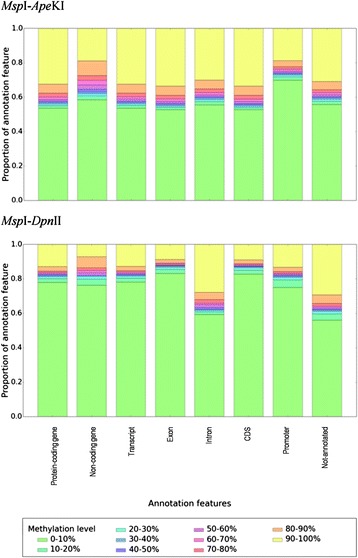
Detected cytosine methylation levels of CG sites in a given annotation within the control line and epiline LR2 (fourth selfing). The proportion of cytosine positions with a particular methylation level bin is displayed as a stacked bar diagram. Ten methylation level bins are considered with an interval of 10%. Proportions of methylation level bins are visualized for different annotation features (*O. sativa* ssp. *indica* ASM465v1.27): protein-coding gene, non-coding gene, transcript, exon, intron, coding DNA sequence (CDS) and promoter (2000-nucleotide region upstream of the TSS)

The available genomic features in *O. sativa* ssp. *indica* allowed to identify their cytosine methylation levels detected by plant-RRBS. The scattered distribution patterns of CG methylation levels in the different annotations were distinguishable between both *Msp*I-*Dpn*II and *Msp*I-*Ape*KI combinations and we analyzed CG methylation levels linked with given genomic features such as promoter or genes in the rice control line and LR2 epiline (Fig. 2). Using the plant-RRBS setup, intronic and not-annotated cytosine positions were found to be rather frequently methylated at CG sites, in contrast to the low frequency of CG methylation detected in the promoters (Fig. 2). *Msp*I-*Ape*KI detected CG methylated sites more frequently in different gene annotations (i.e. protein-coding genes, non-coding genes, transcript, exon and coding DNA sequence (CDS) annotation) as compared with *Msp*I-*Dpn*II. Non-coding genes were less frequently methylated when compared with the other annotation features such as transcript, exon and CDS annotation. Additionally, CG methylated cytosine sites in introns were slightly more frequently detected with *Msp*I-*Ape*KI. The detection of methylation levels in different annotation features gives information about restriction endonuclease combinations enriching to a different extent methylated subfractions of the rice genome.

### Differential cytosine methylation detection

#### Cytosine methylation level homogeneity within five biological replicates per line

The five biological replicates were used to investigate the homogeneity of cytosine methylation within the LR2 epiline. The comparison of cytosine methylation levels within five biological replicates per line and per restriction enzyme combination was performed by applying a 25% methylation level difference threshold as selection between the highest and the lowest detected methylation level at a common position in the biological replicates. The proportion of consistently measured cytosine methylation levels was high, because 96.8% or more were detected at the threshold of less than 25% methylation level difference between the biological replicates in the rice inbred line or the LR2 epiline (Additional file 1: Table S6). It can be concluded that the detected methylation levels of the vast majority of cytosine positions in one sample are consistent with that in the other replicates. In fact, less replicates may be used to investigate biological sample homogeneity.

#### Differential cytosine methylation in the LR2 epiline versus the control line

An important application of methylome profiling is the detection of differential cytosine methylation in certain genomic regions and genomic features between samples, which can assist epigenetic marker detection in breeding programs.

We determined the number of differentially methylated sites and their annotation in the LR2 epiline versus the inbred control line, each analyzed with five biological replicates, for the different cytosine sites (CG, CHG and CHH) by plant-RRBS using the two restriction endonuclease combinations. This allowed to detect more than one thousand differentially methylated positions representing distinct epiallelic states in the LR2 epiline (Table 3). *Msp*I-*Ape*KI resulted in the detection of an order of magnitude more differentially methylated positions (i.e. 1295) in the methylome-profiled rice material as compared with *Msp*I-*Dpn*II (i.e. 142). The number of hypo-methylated cytosine positions detected by *Msp*I-*Ape*KI exceeded that of hyper-methylated positions, whereas *Msp*I-*Dpn*II resulted in the detection of more hyper-methylated positions in the LR2 epiline (Table 3), confirming that the enrichment of specific genome positions depends on the used restriction endonuclease combination. The majority (70%) of differentially methylated sites were CG sites in the profiled LR2 epiline, mainly in not-annotated genomic regions and gene-associated regions (Table 3). The restriction endonuclease combination *Msp*I-*Ape*KI detected a high number of differentially methylated sites in genes, and especially CG sites (Table 3). The 2000 nucleotides upstream of the TSS (i.e. promoters) oftentimes contained more differentially methylated cytosine positions than genes or all annotated regions (Table 3). Plant-RRBS using the rice inbred line and the LR2 epiline and specific restriction endonuclease combinations was therefore focusing on differentially methylated cytosine sites in not-annotated genomic regions, including gene promoters, and within genes. In conclusion, we demonstrate extensive detection of differentially methylated CG sites in the rice LR2 epiline compared with the control line. Integration of methylome with transcriptome will be part of future research.

**Table 3:** Differential cytosine methylation and annotation of the control line versus the LR2 epiline (fourth selfing)

Restriction endonuclease combination	Cytosine site	Number of differentially methylated sites^a^		Number of diff. meth. sites within features			
				Annotated region	Not-annotated region	Gene-associated region	
		Hypo-	Hyper-			Gene	Promoter^b^
*Msp*I-*Ape*KI	CG	541	463	182	822	160	362
	CHG	169	94	28	235	28	81
	CHH	20	8	11	17	4	4
	sum	730	565	221	1074	192	447
*Msp*I-*Dpn*II	CG	29	74	9	94	8	27
	CHG	10	25	0	35	0	11
	CHH	2	2	1	3	1	0
	sum	41	101	10	132	9	38

Cytosine positions, represented by the numbers above, can be counted to the different annotations multiple times

^a^ hypo−/hyper-methylated means a 25% lower/higher methylation level in LR2 compared with the control line

^b^ Promoter is defined as 2000 nucleotides upstream of the TSS and belongs to the not-annotated region in the used genomic features

## Conclusions

A new, performant laboratory protocol and data analysis pipeline of plant-RRBS is reported for cytosine methylome profiling, allowing comparative analysis of multiple samples to detect differential methylation levels at nucleotide resolution in reference genome regions and genomic features. In plants, early RRBS setups using a single restriction endonuclease had a very limited number of covered cytosine positions hindering subsequent comparative analyses [29–31]. Our plant-RRBS protocol enriches for coverage of cytosine positions in reads by applying appropriate restriction endonuclease combinations, that were tested *in silico* at first. It offers broad, genome-dispersed methylation detection by more effective read number usage, as compared with WGBS. Plant-RRBS fulfills the need for an NGS-based cytosine methylome profiling method in large-scale studies in plant science and epigenetic marker-assisted plant breeding. It is applicable to any reference-sequenced plant species, as supported by the promising high *in silico* genome coverage observed for different plant genome sizes with different structural compositions and genomic features. Hence, it will be broadly applicable to profile the methylome of natural or experimental populations, like epilines and epigenetic recombinant inbred lines, in a user-friendly way.

## Methods

### Plant material and growth conditions

A rice pure breeding inbred line (*Oryza sativa ssp. indica*) of Bayer CropScience, named control line, and a derived epiline, named LR2 and selected for a higher EUE, were grown in a growth chamber at Bayer CropScience (Zwijnaarde, Belgium). Starting from a small population (*n* = 180) of a parental rice inbred line, individual plants were selected for lowest cellular respiration and improved EUE (see below). A number of selfings of selected plants followed by *in vitro* assays established the epiline LR2, which was selected over three generations for improved EUE.

The LR2 epiline and the control line were grown in soil in a growth chamber at 26 °C / 21 °C (day / night) for 24 days with a 16-h light/8-h dark regime (light intensity was 300 μmol m^−2^ s^−1^, the relative humidity was kept at 71%). The sample material for the methylome profiling was the fourth leaf of individual plants for each of the five biological replicates per line, which belongs to the fourth selfing generation.

### Assay testing for cellular respiration and EUE analysis

Energy use efficiency, defined as the ratio between energy content and cellular respiration, and energy homeostasis, determined by the crosstalk of molecular networks, are significant components of crop yield stability under varying environmental conditions in the field [22, 25]. Both have an inheritable epigenetic layer of regulation (i.e. cytosine methylation and histone modification) that can be identified and stabilized, resulting in superior agronomical traits in crops [22, 25].

Selection implied a non-destructive assay on an explant per individual in order to identify better performing individuals and was basically done as described for *B. napus* [23] with specific adaptations for rice. Seedlings were *in vitro*-grown in the dark for 10 days on agar in half-strength Murashige and Skoog medium supplemented with 3% (*w*/*v*) sucrose. The first five centimeters above the coleoptiles (primary leaf rolls) were carefully cut in about 0.6-cm segments without damaging the meristem. Seven primary leaf roll explants of each seedling were cultured in the dark for 1 day on callus-inducing medium (Murashige and Skoog medium containing 2% (*w*/*v*) maltose, 3% (*w*/*v*) sorbitol, 2 mg/L 2,4D). The plantlets were cultured in the light to allow outgrowth of the meristem. For each seedling, the cellular respiration of the seven leaf roll explants was quantified by measuring the reduction of triphenyltetrazolium chloride as previously described [44]. The seven explants were transferred to 2 mL of 20 mM 2,3,5-triphenyltetrazolium (TTC) solution in 50 mM K-phosphate buffer (pH 7.4), incubated for 1.5 h in the dark at 26 °C, after which the TTC solution was removed and the explants were washed with water and freeze-thawed. Reduced TTC was extracted with 1 mL ethanol by shaking for about 1.5 h. Absorption of the extract was measured at 485 nm and 663 nm. The absorbance was calculated at OD_485_ due to the reduced TTC (TTC-H): OD_485_ TTC-H = a–(b/c) (a = OD_485_; b = OD_663_; c = constant determined by measuring the absorbance of chlorophyll extract (identical process as described above) at 485 nm and 663 nm → c = OD_663_/ OD_485_). Five to ten seedlings with the lowest cellular respiration were transferred to the greenhouse for seed production by self-fertilization. Both cellular respiration and NAD(P)H content of approximately 35 to 40 seedlings of the obtained progenies were measured as previously described [23]. Lines with the lowest cellular respiration and highest EUE were retained. Three rounds of selfing and testing for the cellular respiration and EUE parameter were sufficient to obtain the LR2 epiline with a distinct cellular respiration and EUE (Additional file 1: Table S1).

### Rice inbred line and epiline used as material for plant-RRBS methylome profiling

Leaf roll explants from about 180 ten-day-old rice seedlings were evaluated for cellular respiration. A number of plantlets, of which the explants had the lowest cellular respiration, were transferred to the greenhouse for seed production by self-fertilization, and repeated three times (Additional file 1: Table S2), resulting in the LR2 epiline with a stable and significantly reduced cellular respiration level in the leaf rolls over at least two consecutive selfing generations (85 to 86%; Additional file 1: Table S2), an NAD(P)H content similar to the control line (100%; Additional file 1: Table S1), an EUE and photorespiration significantly increased to 118% and 106%, respectively, and a significantly reduced (to 80%) leaf respiration rate (Additional file 1: Table S1), that was used for methylome profiling.

### Leaf respiration

For the determination of the leaf respiration, the plants were grown in soil for 4 weeks (temperature: day 26 °C–night 22 °C; light intensity: 400 μMol sec-1 m-2; light regime: 16-h light/8-h dark). The fifth leaf of six plants was harvested and put in 240 mL of buffer (25 mM K-phosphate, pH = 5.8; 2% sucrose; 0.1% Tween20) saturated with oxygen and contained in a closed, 200-mL, scaled bottle. Ten replications per line were performed. The amount of oxygen in the buffer was measured using an optode HQ-portable meter with LDO-electrode (Hach) after four hours of incubation at 24 °C (very gentle shaking). The consumed oxygen was determined by comparing with blank buffer containing no leaf material.

### Plant-reduced representation bisulfite sequencing (plant-RRBS)

#### DNA isolation

Isolation of genomic DNA was performed from an optimal mass of about 60 mg leaf material to obtain a high quality and quantity of genomic DNA, using the Wizard Genomic DNA Purification Kit (Promega) according to manufacturer’s instructions, from five individual plants (biological replicates) of the LR2 epiline or the control line. Genomic DNA isolates were examined for high quality and sufficient quantity by NanoDrop spectrophotometry (concentration, A_260_/A_280_ of approximately 1.8 or more) and gel electrophoresis (high molecular weight, integrity, purity).

### In silico *and double restriction endonuclease digestion*

*In silico* digestion was performed using biopieces v0.48 (www.biopieces.org) of the *A. thaliana* (TAIR 10)*, B. vulgaris* ssp. *vulgaris, B. rapa*, *O. sativa* ssp. *indica* version 9311_BGF_2005, *O. sativa* ssp. *japonica,* and *Z. mays* (B73) nuclear reference genomes [38, 45–48]. The per-base genome coverage by digestion fragments with a length between 150 and 420 bp was expressed relative to the nuclear reference sequence size. About two micrograms of genomic DNA were either digested with firstly *Msp*I (60 U, 37 °C) and secondly *Ape*KI (15 U, 75 °C), or with *Msp*I (60 U, 37 °C) followed by *Dpn*II (30 U, 37 °C) added in two half units portions and in Buffer 3.1 (all obtained from NEB, MA) in a final volume of 60 μL for 20 h each, according to manufacturer’s instructions. Successful digestion was confirmed by gel electrophoresis. For plant-RRBS samples, a smear of fragments of different sizes and no evidence of non-digested, high-molecular mass molecules were observed, indicating successful digestion. Genomic DNA control samples taken along without restriction endonucleases showed a discrete high-molecular mass, indicating the absence of contaminating nucleases and persistent DNA quality despite the incubation procedure.

#### Library preparation and sequencing

Plant-RRBS paired-end libraries for Illumina sequencing were constructed by Alpha Biolaboratory, Inc. Saratoga, CA according to Hsieh (2015) [49] and Pignatta et al. (2015) [50] with modifications. Approximately 300 ng of digested genomic DNA was purified, end repaired, and ligated to custom-synthesized methylated multiplex adapters (Eurofins MWG Operon, Huntsville, AL) according to the manufacturer’s (Illumina, San Diego, CA) instructions. To ensure recovery of shorter plant-RRBS library inserts, the SPRI method with 1.8 x (*v*/v) AMPure XP beads (Beckman Coulter, Brea, CA) was used for cleanup steps throughout the library construction procedure. Adaptor-ligated libraries were subjected to one round of bisulfite conversion with the EZ DNA Methylation-Lightning Kit (Zymo Research Corporation, Irvine, CA) as outlined in the manufacturer’s instructions. Five to ten nanograms of bisulfite-converted libraries were PCR-amplified with the following condition: 2.5 U of ExTaq DNA polymerase (Takara Bio), 5 μL of 10 x Extaq reaction buffer, 25 mM dNTPs, 1 μL of index primers (10 μM) in a 50-μL reaction. The thermocycling conditions were as follows: 95 °C for 3 min and then 12 cycles each of 95 °C for 30 s, 65 °C for 30 s, and 72 °C for 60 s. The enriched libraries were purified twice with 0.8× (*v*/v) AMPure XP beads to remove any adapter dimers. The library quality was assessed by randomly sub-cloning and sequencing 20 to 30 colonies to evaluate proper library construction, bisulfite conversion, and the presence of correct indexes. Final libraries were evaluated by qPCR for library size distribution and quantification in the 2100 Bioanalyzer (Agilent Technologies, Santa Clara, CA). Called peaks ranged between approximately 270 and 540 bp (Additional file 1: Figure S1). The quality-controlled plant-RRBS libraries were then sequenced at the Vincent J. Coates Genomics Sequencing Laboratory at UC Berkeley on an Illumina HiSeq 2500 (PE50–75-100). Bisulfite conversion efficiency rates (approximately 99% or higher) were assessed by calculating cytosine methylation levels in the chloroplast genome.

#### Data processing

All sequencing libraries (ArrayExpress [51] accession numbers: E-MATB-4626 and E-MTAB-5002) were processed by scripts available via doi (10.5281/zenodo.168034) [52] and on GitHub [53], and evaluated for quality using FastQC v0.11.2 [54]. Due to different read lengths of the sequence libraries, reads were trimmed at their 3′ end to a uniform total read length of 50 nucleotides using the FASTX Toolkit v0.0.13 [55]. Adapters were subsequently removed using Trim Galore v0.3.3 with the options --paired --trim1 [56]. By default, Trim Galore also trims nucleotides with a quality lower than 20 (Phred ≥20; base call error rate ≤ 1.0%) prior to adapter trimming and discards reads with a length smaller than 20. Next, the reads were mapped to the reference genome of *O. sativa* ssp. *indica* cultivar 93–11 that was sequenced by the Beijing Genome Institute following a whole-genome shotgun strategy [38] [reference genome: 9311_BGF_2005 PLAZA [57]]. To be able to map reads to the reference genome, a plant-RRBS genome index was created for the defined cutting sites C-CGG,G-CWGC for the *Msp*I-*Ape*KI enzyme combination and C-CGG,-GATC for the *Msp*I-*Dpn*II enzyme combination. Indexing was done using BSseeker v2.0.5 [32], bowtie v2.1.0 [33] and python 2.7.4 [58]. Read alignment was done using BSseeker v2.0.5 and bowtie v2.2.4 with the options --mismatches 2 -r -L 20 -U 500. Mapping quality was assessed using Qualimap v2.1 [34].

The mapping was visualized in Integrative Genomics Viewer (IGV) [59] and igv.js [60]. The per-base genome coverage of the different libraries was calculated using BEDTools genomecov v2.22.0 [35] and expressed relative to the reference genome size. The obtained BAM files were sorted by coordinate using Picard v1.129 [61]. Overlapping reads were clipped using bamUtil v20130118 [36].

PCR duplication information was retrieved from the mapped read files by using the SAM file flag properties and counting the mapped reads with a flag higher than or equal to 1024.

The number of cytosine positions covered per read was retrieved by mapping the reads using the normal procedure (see above), and then performing the calling procedure (see above) with the change that only a single read is necessary for a cytosine position to be called methylated. The intersection of the number of expected reads and the number of sequenced reads was performed using bedtools v2.26.0: a custom bed file was created from the *in silico* predicted fragments which was then intersected with the BAM file of mapped sequenced reads, returning only the fragments that are fully covered in both the *in silico* predictions and sequenced reads.

Methylation level detection was done using BSseeker v2.0.5. The calculation of cytosine site methylation level was performed with Ci/(Ci + Ti) at positions (i) in both DNA strands [26]. Also, the numbers of CG, CHG and/or CHH sites covered by the different libraries were extracted from the BSseeker CGmap.gz output files. Differential methylation was calculated using the R package methylKit v0.5.5 using default parameter settings [37] and R v2.15.1 [62]. Prior to this analysis, the CGmap.gz output files from BSseeker were converted by custom scripting to the BED format required by methylKit. Filtering was done with a threshold of ten informative nucleotides (means C or T) at a cytosine position (BSseeker options: −coverage 10) and normalization of the libraries was performed using standard settings in methylKit (median). Merging of all data was done in so-called ‘unite.txt’ files. Differential methylation of cytosine positions was defined by a threshold of minimum ten informative nucleotides (means C or T) per biological replicate, a pooled number of informative nucleotides for calculation of *p*-values using Fisher’s exact test and q-values <0.01 using the Sliding Linear Model, and percent methylation level differences > │25│% between the in the text indicated comparison pairs of each line calculated per CG, CHG and CHH site using custom scripting [37, 63]. Hypo- / hyper-methylated sites were determined as lower / higher methylation level in the LR2 epiline compared with the control line. A methylation difference analysis between the replicates of each line was performed based on the output of methylKit (i.e. unite files) using > │25│% methylation level differences threshold and custom Python scripting. Annotation of particular nucleotide positions was based on the Oryza_indica.ASM465v1.27.gff3 as obtained from Ensembl Plants [64]. Intronic regions were added using genometools v1.5.4 [65]. Promoter regions were defined as a window of 2000 nucleotides upstream of the TSS. Annotation of covered cytosine positions was done using custom Python scripting. Different features were considered: protein-coding gene, non-coding gene, transcript, exon, intron and CDS. Hence, a site can have multiple annotation features. For each considered contrast, visualization of the average methylation level (as extracted from the unite files) per annotation feature was done by a stacked bar plot using the Python libraries pandas and matplotlib. With non-coding genes possibly having identical child features as protein-coding genes (e.g. typically transcript and exon), child features were discarded when a position belongs to both a non-coding gene and a protein-coding gene. Hereby, the interpretation of protein-coding gene features is not skewed by identical non-coding gene features. One exception was however made for the feature ‘intron’. If a position fell within an intronic region, the intron feature was retained.

#### Whole-genome bisulfite sequencing (WGBS)

For genome-wide DNA methylome analysis by whole-genome bisulfite sequencing (WGBS), the isolation of genomic DNA was performed from 12 pooled fourth leaves of individual plants for the control line and for the LR2 epiline, which belong to the fourth selfing generation, using the CTAB (cetyltrimethyl/ammonium bromide) standard protocol [66]. Genomic DNA isolates were examined for high quality and sufficient quantity by NanoDrop spectrophotometry (concentration, A_260_/A_280_ of approximately 1.9 or more) and gel electrophoresis (high molecular weight, integrity, purity). For WGBS, paired-end bisulfite sequencing libraries were constructed by Alpha Biolaboratory, Inc. Saratoga, CA as described previously [67] with modifications. About 300 ng of genomic DNA was fragmented by sonication, end repaired and ligated to custom-synthesized methylated adapters (Eurofins MWG Operon, Huntsville, AL) according to the manufacturer’s (Illumina, San Diego, CA) instructions for genomic DNA library construction. Adaptor-ligated libraries were subjected to two successive treatments of sodium bisulfite conversion using the EpiTect Bisulfite kit (Qiagen, Hilden, Germany) as outlined in the manufacturer’s instructions. One quarter of the bisulfite-converted libraries was PCR amplified using the following conditions: 2.5 U of ExTaq DNA polymerase (Takara Bio), 5 μL of 10 x Extaq reaction buffer, 25 μM dNTPs, 1 μL primer 1.1, 1 μl primer 2.1 in a 50-μL reaction. The thermocyling conditions were as follows: 95 °C for 3 min, then 12–14 cycles of 95 °C for 30 s, 65 °C for 30 s and 72 °C for 60 s. The enriched libraries were purified twice with SPRI method using 0.8 x *v*/v AM-Pure beads (Beckman Coulter, Brea, CA) prior to quantification with a Bioanalyzer (Agilent Technologies, Santa Clara, CA). Sequencing on the Illumina platform (SE100 runs) was performed at the Vincent J. Coates Genomic Sequencing Laboratory at UC Berkeley. The WGBS data analysis was performed with the same workflow used for the plant-RRBS data, with the exception that no cut-site specific indices were build. Genome coverage and cytosine coverage were determined in the same manner as for the plant-RRBS data. Bisulfite conversion efficiency rates (approximately 99%) were assessed by calculating cytosine methylation levels in the chloroplast genome.

## Additional file

Additional file 1: Additional files may be found in the online version of this article: **Figure S1.** Examples of electropherograms of library quality. **Figure S2.** Normalized overlap percentage of number of detected methylated sites between different RRBS samples per restriction enzyme and group. **Figure S3.** Integrative Genomics Viewer (IGV) screenshot of a representative genome region of RRBS and WGBS coverage data and mapped reads. **Figure S4.** Cytosine coverage in representative RRBS and WGBS samples. **Figure S5.** Annotation of methylated and common cytosine positions located on chromosomes between the biological replicates of different restriction endonuclease combinations and the control line and the LR2 epiline LR2 of selfing generation 4. **Table S1.** Physiological properties of the rice LR2 epiline versus the control inbred line (%). **Table S2.** Cellular respiration in the LR2 epiline (% versus the control inbred line) during consecutive selfings of the epilines. **Table S3.** Percentage of PCR duplicates in the input data per sample. **Table S4.** Read preprocessing and mapping quality. **Table S5.** Intersection of *in silico* fragments and mapped reads (%). **Table S6.** Intra-line similarity between biological replicates per line of selfing generation 4 based on the methylation level difference of the cytosine sites CG, CHG and CHH detected in the replicates. (DOCX 1363 kb)

## Abbreviations

BAM: Binary Alignment/Map
BED: Browser Extensible Data
Bisulfite: Sodium hydrogen sulfite
CDS: Coding DNA sequence
CMT3: Chromomethylase 3
Control: Pure breeding rice line
DDM1: Decrease in DNA methylation 1
DRD1: Defective in RNA-directed DNA methylation 1
DRM1/2: Domains rearranged methylase 1/2
EUE: Energy use efficiency
GBS: Genotyping by sequencing
Intersection: Common set
Jaccard similarity coefficient: Jaccard index
MET1: DNA methyltransferase 1
NAD(P)H: Reduced Nicotinamide adenine dinucleotide phosphate
NGS: Next-generation DNA sequencing
Plant-RRBS: Plant reduced representation bisulfite sequencing
qPCR: Quantitative PCR
RRBS: Reduced representation bisulfite sequencing
S4: Selfing generation 4
SPRI: Solid-phase reversible immobilization
TE: Transposable element
TSS: Transcription start site
TTC: 2,3,5-triphenyltetrazolium
TTC-H: Reduced TTC
Union: Total set
WGBS: Whole-genome bisulfite sequencing
