# Supplementary material for: Plant-RRBS, a bisulfite and next-generation sequencing-based methylome profiling method enriching for coverage of cytosine positions
Source: BMC Plant Biol. 2017 Jul 6;17:115. doi: 10.1186/s12870-017-1070-y (PMC5501559; doi:10.1186/s12870-017-1070-y)
Supplement: Supplementary file 1 — Additional files may be found in the online version of this article: Figure S1. Examples of electropherograms of library quality. Figure S2. Normalized overlap percentage of number of detected methylated sites between different RRBS samples per restriction enzyme and group. Figure S3. Integrative Genomics Viewer (IGV) screenshot of a representative genome region of RRBS and WGBS coverage data and mapped reads. Figure S4. Cytosine coverage in representative RRBS and WGBS samples. Figure S5. Annotation of methylated and common cytosine positions located on chromosomes between the biological replicates of different restriction endonuclease combinations and the control line and the LR2 epiline LR2 of selfing generation 4. Table S1. Physiological properties of the rice LR2 epiline versus the control inbred line (%). Table S2. Cellular respiration in the LR2 epiline (% versus the control inbred line) during consecutive selfings of the epilines. Table S3. Percentage of PCR duplicates in the input data per sample. Table S4. Read preprocessing and mapping quality. Table S5. Intersection of in silico fragments and mapped reads (%). Table S6. Intra-line similarity between biological replicates per line of selfing generation 4 based on the methylation level difference of the cytosine sites CG, CHG and CHH detected in the replicates. (DOCX 1363 kb) [file 12870_2017_1070_MOESM1_ESM.docx]

**Supporting Information**

**Plant-RRBS, a bisulfite and next-generation sequencing-based methylome profiling method enriching for coverage of cytosine positions**

**Martin Schmidt^1,2^, Michiel Van Bel^1,2,^ , Magdalena Woloszynska^1,2,^ , Bram Slabbinck^1,2^, Cindy Martens^3^, Marc De Block^3^, Frederik Coppens^1,2^ and Mieke Van Lijsebettens^1,2,*^**

*^1^Department of Plant Biotechnology and Bioinformatics, Ghent University, 9052 Ghent, Belgium,*

*^2^VIB Center for Plant Systems Biology, 9052 Ghent, Belgium and*

*^3^Bayer Cropscience N.V., Innovation Center, Technologiepark 38, 9052 Ghent, Belgium*

^*^For correspondence (Tel. +32 (0)9 331 39 70; Fax +32 (0)9 331 38 09; [milij@psb.ugent.be](mailto:milij@psb.ugent.be)).

*** Corresponding author:**

Mieke Van Lijsebettens

Department of Plant Biotechnology and Bioinformatics / Department of Plant Systems Biology

Ghent University / VIB

Technologiepark 927

B - 9052 Ghent, Belgium

Tel: + 32 (0)9 331 39 70

Fax: + 32 (0)9 331 38 09

E-mail: [milij@psb.ugent.be](mailto:milij@psb.ugent.be)


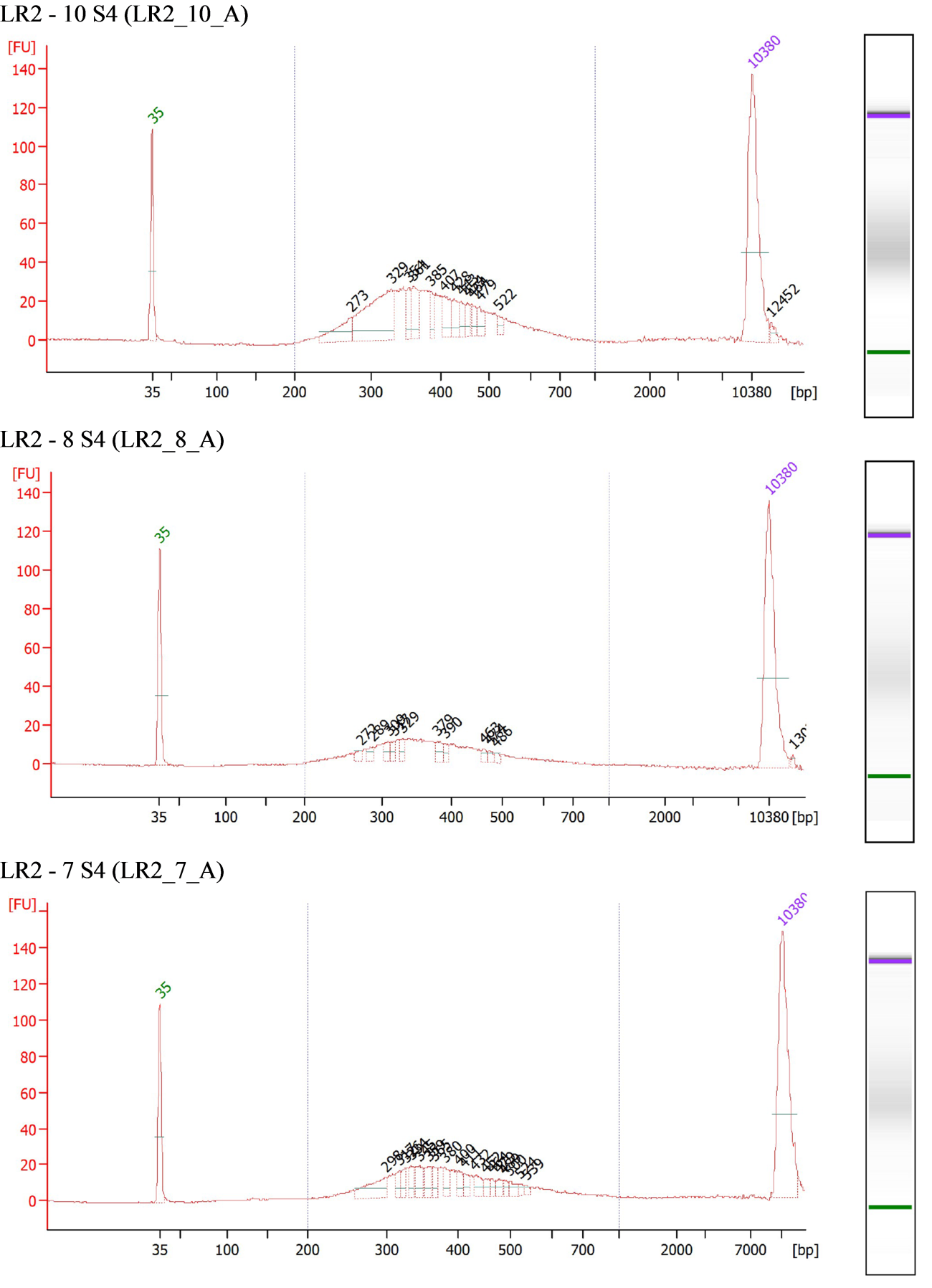


**Figure S1.** Examples of electropherograms of library quality. Name scheme: line – individual plant number (7, 8, 10) of selfing generation 4 (S4) followed by library base prefix (provided by Dr. I-Chen Chen from Alpha Biolaboratory, Inc, US).

**Figure S2.** Normalized overlap percentage of number of detected methylated sites between the different RRBS samples, per restriction enzyme (*Msp*I-*Ape*KI vs. *Msp*I-*Dpn*II) and per group (Control vs. LR). The percentages are stacked, so the total percentage of detected methylated sites by 3 or more samples is ~80% for *Msp*I-*Ape*KI control.


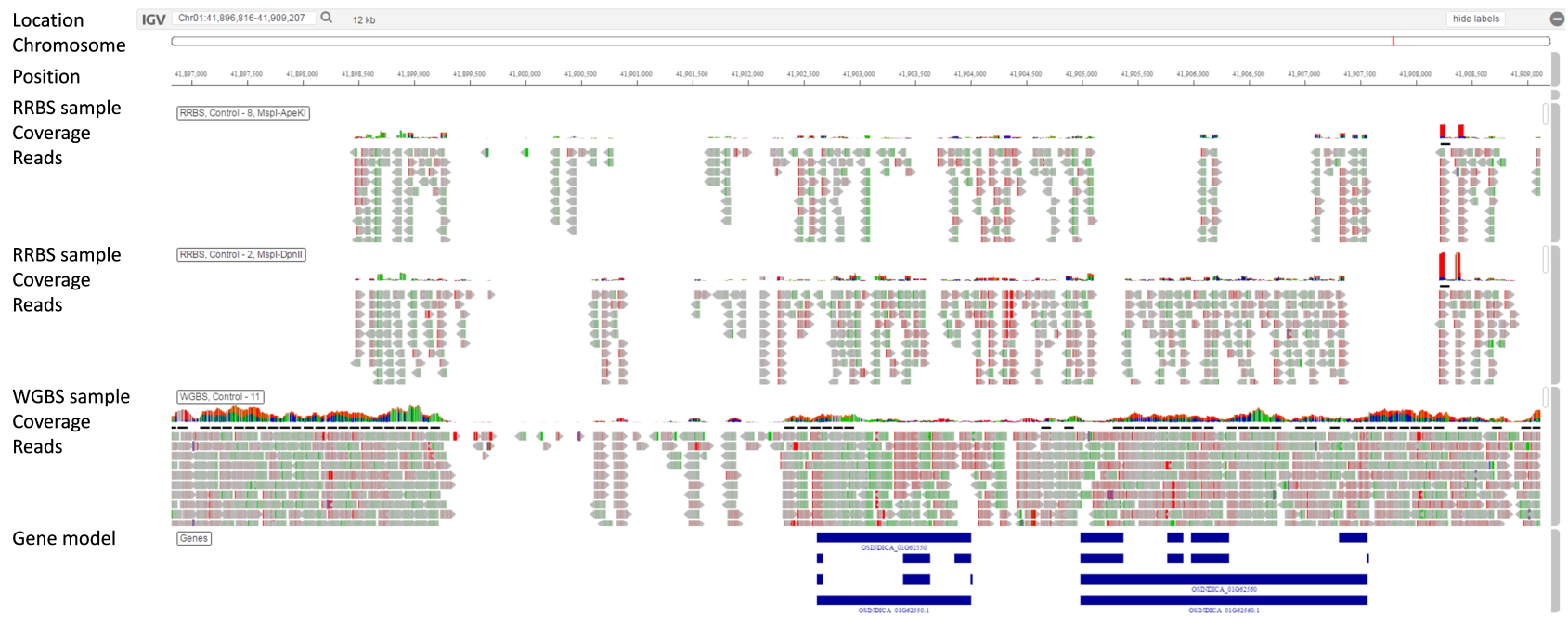


**Figure S3.** Integrative Genomics Viewer (IGV) screenshot of a representative genome region of RRBS and WGBS coverage data and mapped reads. The chromosome location is shown on the top, followed by a chromosome scheme and the indication of the location as a red line. The scale shows the position on the chromosome in more detail. Below, representative RRBS and WGBS samples are shown each with coverage as histogram and the mapped reads as grey arrows. Additional colors are default settings in IGV. The first 10 reads are displayed by IGV (default) and the display of further reads is cropped as indicated by the scroll bar to the right. On the bottom, the gene model is displayed.


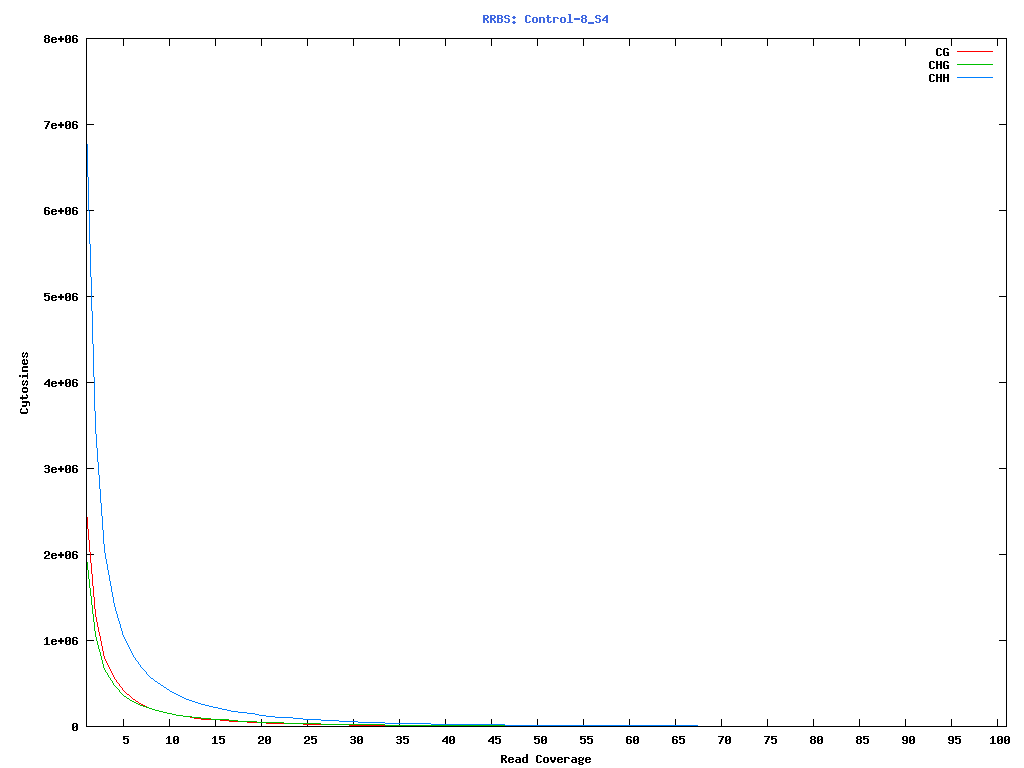


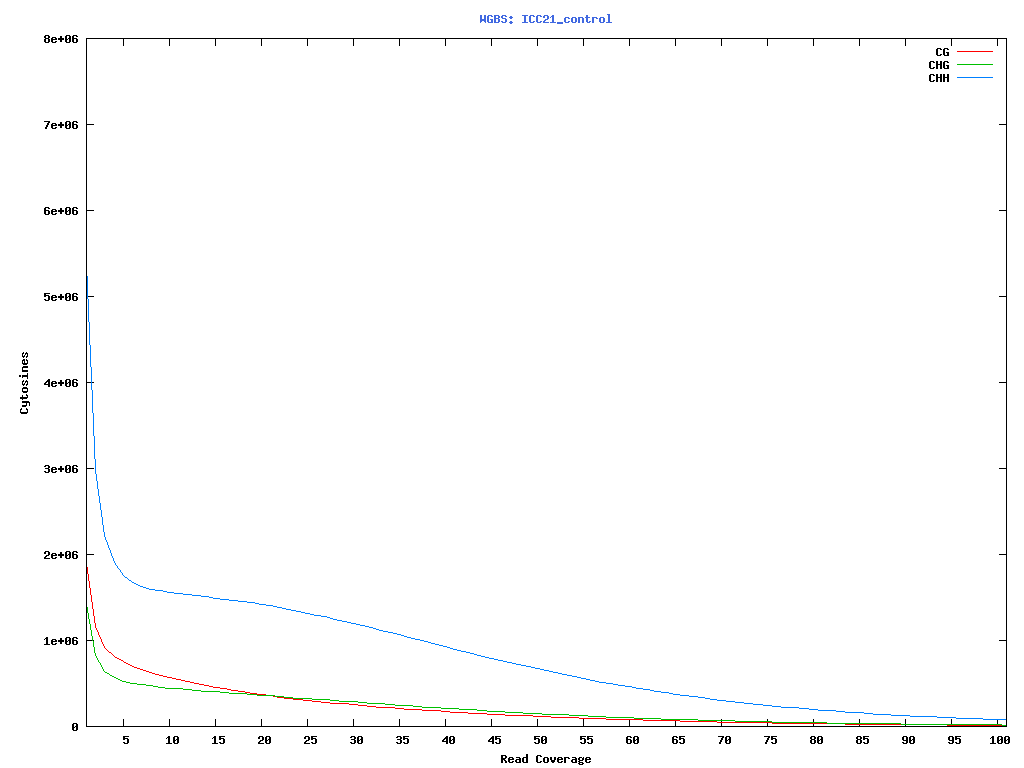


**Figure S4.** Cytosine coverage in representative RRBS (top) and WGBS (below) samples. Cytosine sites are color coded.

**
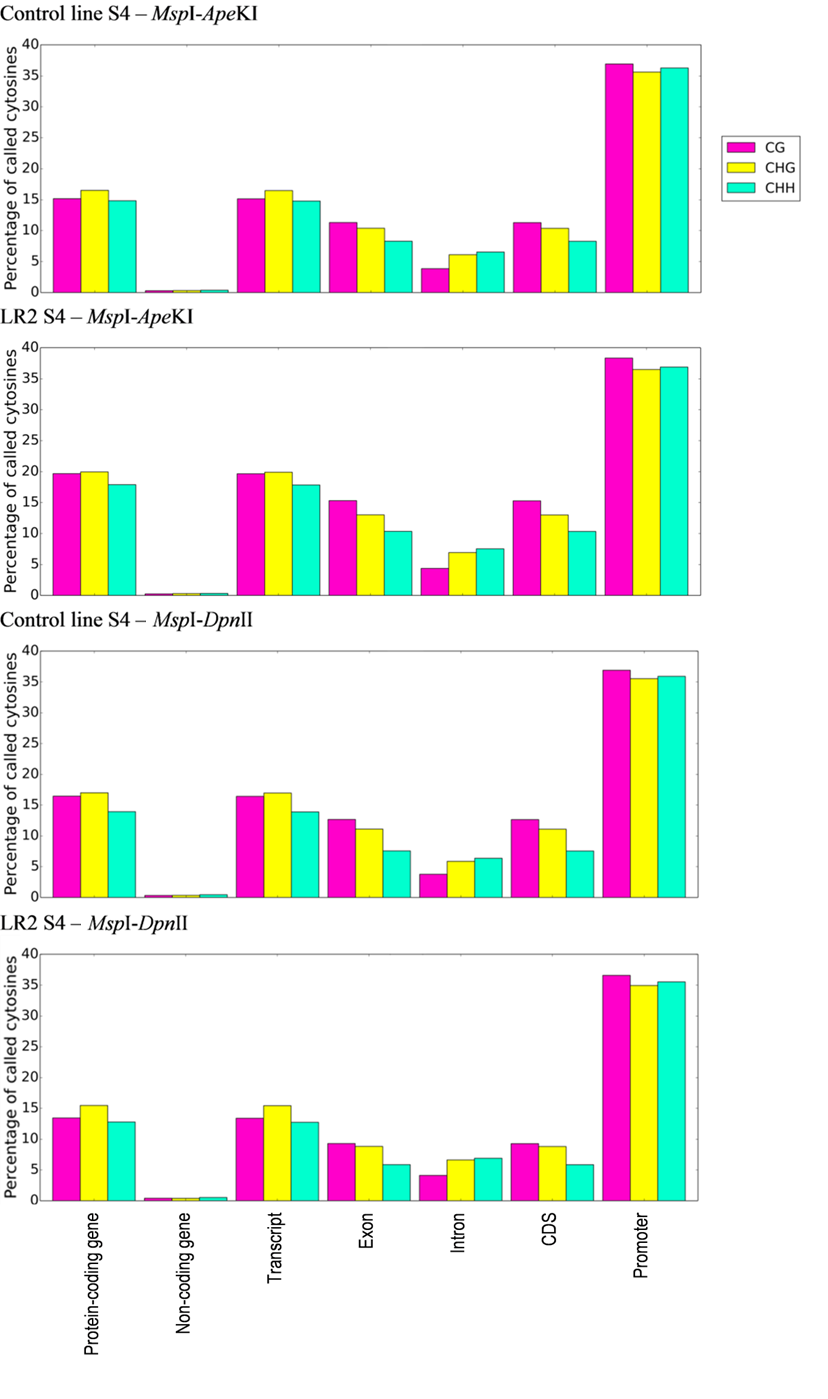
Figure S5.** Annotation of detected and common cytosine positions located on chromosomes between the biological replicates of different restriction endonuclease combinations and the control line and the LR2 epiline of selfing generation 4. The percentages are calculated as number of cytosine positions with a particular genomic feature * 100% / all positions per CG, CHG and CHH sites. Annotations are based on the *O. sativa* ssp. *indica* annotation ASM465v1.27: the protein-coding gene, non -coding gene, transcript, exon, intron, CDS (coding sequence) and promoter features are considered. A promoter is considered as the 2,000‑nt region upstream of the transcription start site. Filtering was done for detected cytosine positions, using a threshold for sufficiently mapped cytosine positions in the reference genome by at least ten informative nucleotides (means C or T).

**Table S1.** Physiological properties of the rice LR2 epiline versus the control inbred line (%).

| **Line** | ***In vitro* assays** | | | | **Soil** | |
| --- | --- | --- | --- | --- | --- | --- |
|  | Cellular respiration (S4) | NAD(P)H content (S4) | Energy use efficiency (S4)^a^ | Photorespiration (S4) | | Leaf respiration (S5) |
| Control | 100 | 100 | 100 | 100 | | 100 |
| LR2 | 85*** | 100 | 118** | 106 | | 80* |

ANOVA with Dunnett’s post hoc multiple comparison test, * P < 0.05, ** P < 0.01, and *** P < 0.001.

^a^ NAD(P)H/cellular respiration.

Material from selfing generation 4 (S4) or 5 (S5) is indicated.

**Table S2.** Cellular respiration in the rice LR2 epiline (%) versus the control inbred line during consecutive selfings of the epiline.

| **Line** | **Selfing (S) generations** | | | | |  |
| --- | --- | --- | --- | --- | --- | --- |
|  | **Selection for cellular respiration** | | | **Upscaling (no selection)** | | |
|  | **S1^a^** | **S2^a^** | **S3^b^** | **S4^b^** | **S5^b^** | |
| Control | 100 | 100 | 100 | 100 | 100 | |
| LR2 | 95 | 87 | 86*** | 85*** | 86*** | |

^a^ No statistics done: only one repetition (limited seed availability).

^b^ Three repetitions/assay; 35 to 40 seedlings/repetition, ANOVA with Dunnett’s post hoc multiple comparison test, ^***^ *P* < 0.001, versus control line (%).

**Table S3.** Percentage of PCR duplicates in the input data per sample.

| DataType | Name | FileName | PCR Duplicates |
| --- | --- | --- | --- |
| *Msp*I-*Dpn*II | Control-1 S4 | R01_ATCACG_L005 | 0.368853% |
| *Msp*I-*Dpn*II | Control-2 S4 | R02_CGATGT_L003 | 0.473839% |
| *Msp*I-*Dpn*II | Control-3 S4 | R03_TTAGGC_L006 | 0.667169% |
| *Msp*I-*Dpn*II | Control-4 S4 | R04_TGACCA_L003 | 0.722764% |
| *Msp*I-*Dpn*II | Control-5 S4 | R05_ACAGTG_L007 | 0.597728% |
| *Msp*I-*Dpn*II | LR2-1 S4 | R11_ATCACG_L007 | 0.222455% |
| *Msp*I-*Dpn*II | LR2-2 S4 | R12_CGATGT_L006 | 0.230479% |
| *Msp*I-*Dpn*II | LR2-3 S4 | R13_TTAGGC_L007 | 0.080359% |
| *Msp*I-*Dpn*II | LR2-4 S4 | R14_TGACCA_L006 | 0.186213% |
| *Msp*I-*Dpn*II | LR2-6 S4 | R15_ACAGTG_L006 | 0.245962% |
| *Msp*I-*Ape*KI | Control-6 S4 | R06_GCCAAT_L005 | 0.529878% |
| *Msp*I-*Ape*KI | Control-7 S4 | R07_CAGATC_L003 | 0.71799% |
| *Msp*I-*Ape*KI | Control-8 S4 | R08_ACTTGA_L005 | 0.683113% |
| *Msp*I-*Ape*KI | Control-9 S4 | R09_GATCAG_L007 | 0.546088% |
| *Msp*I-*Ape*KI | Control-10 S4 | R10_TAGCTT_L005 | 0.689001% |
| *Msp*I-*Ape*KI | LR2-6 S4 | ICI002A_ATCACG_L002 | 0.6361% |
| *Msp*I-*Ape*KI | LR2-7 S4 | ICI001A_CGATGT_L001 | 0.673563% |
| *Msp*I-*Ape*KI | LR2-8 S4 | ICI002B_TTAGGC_L002 | 0.699294% |
| *Msp*I-*Ape*KI | LR2-9 S4 | ICI001B_TGACCA_L001 | 0.580727% |
| *Msp*I-*Ape*KI | LR2-10 S4 | ICI001C_ACAGTG_L001 | 0.627014% |
| WGBS | ICC21 | ICC21_control | 0.764089% |
| WGBS | ICC26 | ICC26_LR5007 | 0.650766% |

**Table S4.** Read preprocessing and mapping quality.

| **Line with biological replicates^a^** | **Library (base prefix)** | **Restriction endonuclease combination** | **Read length (paired end)** | **Total reads** | **Mean mapping quality (phred scale)^b^** |
| --- | --- | --- | --- | --- | --- |
| **Plant-RRBS** |  |  |  |  |  |
| Control - 1 | control_1_B | *Msp*I-*Dpn*II | 50 | 42,757,316 | 51.60 |
| Control - 2 | control_2_B | *Msp*I-*Dpn*II | 50 | 71,719,086 | 51.37 |
| Control - 3 | control_3_C | *Msp*I-*Dpn*II | 100 | 43,893,046 | 49.71 |
| Control - 4 | control_4_B | *Msp*I-*Dpn*II | 50 | 71,831,168 | 50.39 |
| Control - 5 | control_5_C | *Msp*I-*Dpn*II | 100 | 17,791,806 | 49.04 |
| LR2 - 1 | LR2_1_B | *Msp*I-*Dpn*II | 50 | 161,579,422 | 54.54 |
| LR2 - 2 | LR2_2_B | *Msp*I-*Dpn*II | 50 | 89,894,718 | 54.24 |
| LR2 - 3 | LR2_3_C | *Msp*I-*Dpn*II | 100 | 14,693,298 | 50.39 |
| LR2 - 4 | LR2_4_B | *Msp*I-*Dpn*II | 50 | 80,867,382 | 55.15 |
| LR2 - 5 | LR2_5_B | *Msp*I-*Dpn*II | 50 | 52,036,812 | 52.33 |
| Control - 6 | control_6_B | *Msp*I-*Ape*KI | 50 | 67,574,988 | 39.37 |
| Control - 7 | control_7_B | *Msp*I-*Ape*KI | 50 | 67,804,782 | 38.48 |
| Control - 8 | control_8_B | *Msp*I-*Ape*KI | 50 | 61,662,938 | 38.20 |
| Control - 9 | control_9_C | *Msp*I-*Ape*KI | 100 | 24,427,868 | 37.66 |
| Control - 10 | control_10_B | *Msp*I-*Ape*KI | 50 | 72,461,786 | 39.27 |
| LR2 - 6 | LR2_6_A | *Msp*I-*Ape*KI | 75 | 42,920,524 | 39.06 |
| LR2 - 7 | LR2_7_A | *Msp*I-*Ape*KI | 75 | 43,676,236 | 38.32 |
| LR2 - 8 | LR2_8_A | *Msp*I-*Ape*KI | 75 | 56,587,898 | 38.89 |
| LR2 - 9 | LR2_9_A | *Msp*I-*Ape*KI | 75 | 44,902,896 | 39.95 |
| LR2 - 10 | LR2_10_A | *Msp*I-*Ape*KI | 75 | 48,669,508 | 38.94 |
| **WGBS** |  |  |  |  |  |
| Control - 11 | LR2_ 11_D | **-** | 100  (single end) | 383,993,330 | **-** |
| LR2 – 11 | Control_11_D | **-** | 100  (single end) | 337,468,167 | **-** |

^a^ Name scheme: line – individual plant number (1 – 11) from selfing generation 4 (S4).

^b^ The mean mapping quality is denoted in the quality phred scale and gives the probability of having an incorrect read alignment.

**Table S5.** Intersection of *in silico* fragments and mapped reads (%).

| **Line with biological replicates^a^** | **Mapped reads from RRBS (BAM files)** | **In silico fragments intersected with mapped reads** | **Intersection of *in silico* fragments and mapped reads (%)** |
| --- | --- | --- | --- |
| Control-1 | 19986170 | 13732167 | 68.71 |
| Control-2 | 35784590 | 25952903 | 72.53 |
| Control-3 | 25781002 | 19618024 | 76.09 |
| Control-4 | 41528034 | 32146404 | 77.41 |
| Control-5 | 10770298 | 8370681 | 77.72 |
| Control-6 | 29929474 | 21059824 | 70.36 |
| Control-7 | 36246178 | 26913839 | 74.25 |
| Control-8 | 29358084 | 22171509 | 75.52 |
| Control-9 | 11503390 | 7931491 | 68.95 |
| Control-10 | 37575308 | 24643281 | 65.58 |
| LR2-1 | 69139508 | 45117730 | 65.26 |
| LR2-2 | 37952220 | 25103151 | 66.14 |
| LR2-3 | 5982228 | 3871905 | 64.72 |
| LR2-4 | 31785462 | 20879938 | 65.69 |
| LR2-5 | 21750642 | 14782903 | 67.97 |
| LR2-6 | 23421470 | 15535814 | 66.33 |
| LR2-7 | 25192822 | 18474916 | 73.33 |
| LR2-8 | 32113912 | 23320675 | 72.62 |
| LR2-9 | 21940144 | 12032040 | 54.84 |
| LR2-10 | 28468818 | 20651057 | 72.54 |

**Table S6.** Intra-line similarity between biological replicates per line of selfing generation 4 based on the methylation level difference of the cytosine sites CG, CHG and CHH detected in the replicates. The numbers of cytosine sites with a lower and higher methylation difference than the defined threshold within the replicates are provided as well as a percentage for the former.

| **Restriction endonuclease combination** | **Line** | **Number of sites with methylation difference < 25 %** | **Number of sites with methylation difference > 25 %** | **Percentage of sites with methylation difference < 25 % (%)^a^** |
| --- | --- | --- | --- | --- |
| *Msp*I-*Ape*KI | Control | 722,389 | 15,616 | 97.9 |
|  | LR2 | 714,536 | 23,469 | 96.8 |
| *Msp*I-*Dpn*II | Control | 55,081 | 813 | 98.5 |
|  | LR2 | 54,839 | 1,055 | 98.1 |

^a^ The number of sites with a methylation level difference lower than 25 % / common positions * 100%.
